# Congenital Mesoblastic Nephroma: A Case Report

**DOI:** 10.31729/jnma.7979

**Published:** 2023-03-31

**Authors:** Ankita Simkhada, Ramesh Paudel, Nisha Sharma

**Affiliations:** 1 Department of Pathology, Maharajgunj Medical Campus, Maharajgunj, Kathmandu, Nepal

**Keywords:** *case reports*, *congenital mesoblastic nephroma*, *kidney neoplasms*, *nephrectomy*

## Abstract

Congenital mesoblastic nephromas are rare renal tumours that are encountered in paediatric age group. A term female neonate at the end of first week of life presented with bilateral lower limb swelling. On radiological evaluation, ultrasonography revealed an intra-abdominal mass which was managed with radical nephroureterectomy. Histopathological examination confirmed a diagnosis of congenital mesoblastic nephroma of mixed subtype.

## INTRODUCTION

Wilm's tumour accounts for approximately 90% of paediatric renal tumours and is the most common renal neoplasm in children between one to four years of age.^1^ Renal tumours are rare in children less than three months of age. Among neonates, congenital mesoblastic nephroma (CMN), is more common.^2^ CMN can be associated with antenatal complications such as polyhydramnios, preterm labour and premature delivery.^3^ It is often recognized in the newborn who presents with an abdominal mass. Radical nephrectomy is the treatment of choice with adjuvant chemotherapy recommended for the more aggressive cellular or mixed variants.^4^ We report a rare case of congenital mesoblastic nephroma in a term neonate born via normal vaginal delivery.

## CASE REPORT

A term female neonate at ten days of her life was taken to a peripheral health facility with complaints of bilateral swelling of lower limbs. She weighed 2500 g at birth and was reported to have cried immediately after birth. During the antenatal period, the mother had regular antenatal checkups and had an uneventful pregnancy.

At the health centre, ultrasonography of lower limbs showed findings suggestive of cellulitis and abdominal ultrasonography showed a large exophytic mass measuring 57x54 mm in the upper pole of the right kidney. She was then referred to a tertiary care centre for further management.

Computed Tomography (CT) scan of the chest and abdomen was done which revealed a heterogeneously enhancing, well defined soft tissue density space occupying lesion in the right abdominal cavity arising from the right kidney, extending from the subhepatic region to the pelvic brim. The tumour was seen crossing the midline and displacing the bowel loops towards the left abdominal cavity. Areas of calcification were not noted. Findings from bilateral lung and mediastinum were normal.

With these findings, a provisional diagnosis of Wilm's tumour was made and oncology consultation was done following which she was planned for a radical nephrectomy. Preoperative blood investigations including liver, renal function tests and complete blood counts were all normal. She underwent radical right nephroureterectomy, the mass was resected as a whole and sent to the Department of Pathology at another tertiary care centre for histopathological examination.

**Figure 1:**
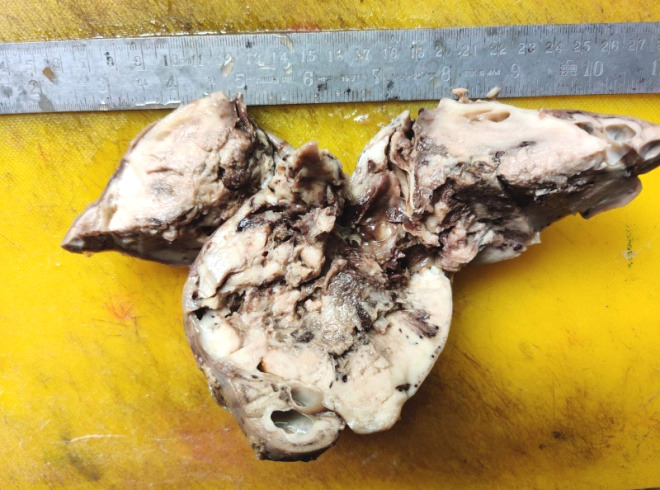
Gross section of resected right kidney showing a grey white solid tumour occupying the whole kidney with focal areas of cystic change.

On gross examination, the right kidney measured 11x10x9 cm which showed a mass measuring 8x8x7 cm which extended from the upper up to the lower pole of the kidney. On the cut section, the mass was solid, grey white with focal cystic areas. The right ureter, measuring 3.5x0.5x0.5 cm was sent in a separate container and was grossly unremarkable (Figure 1).

**Figure 2:**
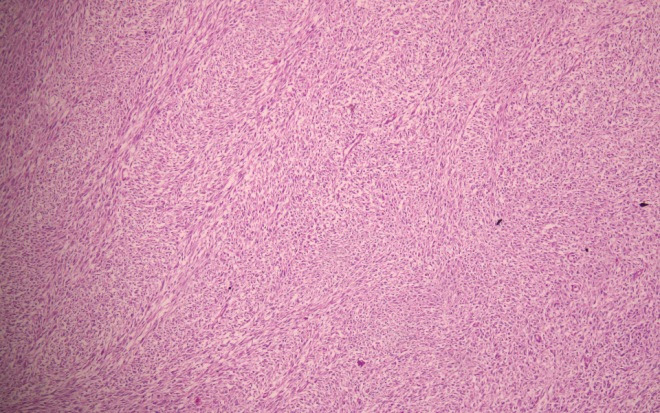
Fascicles of spindle shaped cells are observed (left) along with more cellular areas with ovoid cells (right). (Hematoxylin and eosin stain x 100).

On microscopic examination, the tumour was arranged predominantly in sheets and consisted of highly cellular areas with densely packed tumour cells as well as focal areas showing interlacing fascicles of tumour cells (Figure 2).

**Figure 3:**
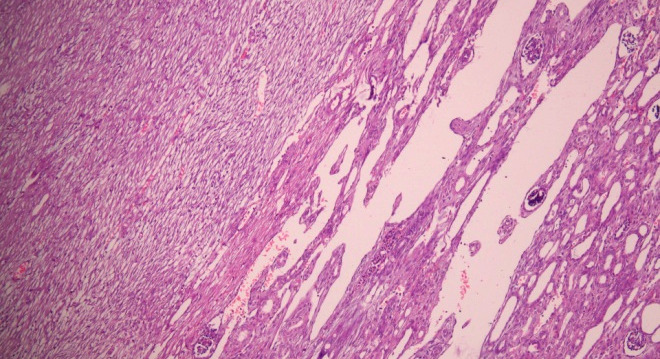
The tumour (left) kidney (right) interface is seen. (Hematoxylin and eosin stain x 100).

Mitotic figures were 5/10 high-power field (HPF). Tumour was limited to the kidney with intact overlying renal capsule and hilar vessels as well as the ureter were free of tumour. Focally, small area of normal renal parenchyma showing glomeruli and tubules was also seen, adjacent to the tumor (Figure 3).

**Figure 4:**
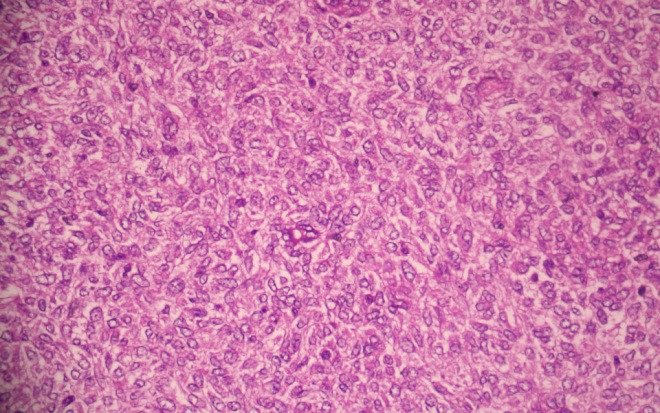
Tumour cells with scant cytoplasm, oval vesicular nuclei and inconspicuous nucleoli. (Hematoxylin and eosin stain x 400).

Tumour cells had a scant amount of eosinophilic cytoplasm with oval to spindled vesicular nuclei and inconspicuous to small prominent nucleoli in some of the cells (Figure 4).

A diagnosis of congenital mesoblastic nephroma, mixed subtype was made. She was discharged seven days post-surgery with stable vitals. Follow up at the paediatric surgical outpatient department was advised, with a plan to start adjuvant chemotherapy with actinomycin D and vincristine on follow up.

One year post surgery, the child is doing well with no signs of recurrence on follow up ultrasonography. However, as the condition of the child has improved and the parents are happy about the progress, after discussion with the treating clinicians, they decided to skip chemotherapeutic treatment.

## DISCUSSION

CMN is an uncommon mesenchymal neoplasm that represents 2-4% of all paediatric renal tumors.^5^ It is the most common congenital renal tumour.^2^ Around 90% cases are diagnosed within the first year of life.^5^ Neonates most commonly present with an intraabdominal mass.^2^ Antenatal complications such as polyhydramnios, preterm labour and premature delivery may also be associated in around 71% cases.^3^ Paraneoplastic syndromes including hypertension and hypercalcemia, though uncommon, have also been reported.^2^ Increased renin synthesis by the tumor cells is thought to be the cause of hypertension whereas secretion of parathormone like proteins is responsible for hypercalcemia.^2^

Due to routine use of ultrasonography, most cases are picked up during the antenatal period and the management plan initiated accordingly. In our case, tumour was diagnosed during the postnatal period when the child presented with lower limb swelling and ultrasonographic investigation for the same incidentally revealed a right sided renal mass.

Congenital mesoblastic nephroma are low grade fibroblastic neoplasms that arise from the infantile renal sinus. These are more common than Wilm's tumor during the neonatal period however the two entities may not be distinguishable radiologically. On imaging, cases of classic CMN appear as homogeneous solid mass with "ring" sign of concentric hypoechoic and hyperechoic rims surrounding the tumour.^6^ Cellular variant in contrast appear heterogeneously enhancing with fluid filled spaces representing haemorrhage, necrosis and cystic changes.^6^

On macroscopic examination, classic CMN exhibit a solid firm grey white tumour with whorled appearance in contrast to the cellular variants in which the mass is soft, cystic with areas of haemorrhage and necrosis. Among the three histological variants of CMN, cellular variant is most common, representing 66% of cases.^5^ The classic variant accounts for 24% of the cases and the mixed subtype is least common, accounting for 10% cases.^7^ Morphologically, tumours in classic CMN resemble infantile myofibromatosis and are composed of fascicles of fibroblastic cells with tapered nuclei, eosinophilic cytoplasm and a low mitotic activity.^8^

In contrast, cellular variant of CMN morphologically has a greater cellularity and resembles infantile fibrosarcoma.^8^ Tumour cells arranged in ill formed fascicles give rise to sheet-like pattern of tumour cells. Tumour cells are plump, ovoid, with vesicular nuclei and moderate amount of cytoplasm. Mitotic activity is usually high and areas of necrosis and haemorrhage may be seen.^8^ Mixed CMN demonstrates features of both cellular as well as classic CMNs. In keeping with this, our case also showed tumour cells composed of dense areas of ovoid shaped cells in addition to spindle shaped cells arranged in fascicles. Tumour cells in CMN are positive for vimentin, desmin and actin but negative for CD34 and epithelial markers, however diagnosis is primarily based on morphology.^9^

Recent genetic studies have shown differences between classic and cellular variants of CMN. The cellular variant shows a translocation (12;15) (p13;q25) with formation of ETV6-NTRK3 gene which is not seen in the cellular variant.^10^

The treatment of choice for CMN is radical nephrectomy. Nephrectomy alone is usually sufficient and only around 5% patients develop recurrence that is mainly related to the completeness of tumour resection.^11^ Chances of recurrence are higher in patients are older than 3 months of age with the cellular variant of CMN.^4^ Recurrences are also common when surgical margins are positive or if there has been an intraoperative tumour rupture.^4^ All of these cases as well as cases of relapse are eligible to receive adjuvant chemotherapy.^4^ Combinations of vincristine, cyclophosphamide and doxorubincin (VCD), vincristine, doxorubicin and actinomycin D (VDA) have been successfully used. ^4^

The differential diagnosis for renal tumours in paediatric population commonly include Wilm's tumour, congenital mesoblastic nephroma, clear cell sarcoma of kidney (CSSK), ossifying renal tumour of kidney (ORTI) and malignant rhabdoid tumour.^2^ Neuroblastomas can also be a differential diagnosis for congenital tumours and may invade the kidney or rarely even arise from the renal parenchyma. In cases of CMN, rarity of this tumour and unfamiliarity with cases can pose a diagnostic difficulty. Radiological features and morphological findings must be used synergistically to reach a diagnosis and the pathologist must always keep in mind the possibility of CMN in case of a renal tumour in neonates.

CMN are the most common congenital renal neoplasms which are mostly diagnosed in the first year of life. These are low grade neoplasms that carry excellent prognosis with radical nephrectomy. Surgical management is adequate for classic variants however adjuvant chemotherapy is recommended for the more aggressive cellular and mixed variant of CMN.
